# Comprehensive analysis of clinical significance of stem-cell related factors in renal cell cancer

**DOI:** 10.1186/1477-7819-9-121

**Published:** 2011-10-07

**Authors:** Yongchao Liu, Changcun Zhang, Jie Fan, Lei Xiao, Bingde Yin, Libin Zhou, Shujie Xia

**Affiliations:** 1 Department of Urology, Shanghai First People's Hospital, School of Medicine, Shanghai Jiaotong University, Shanghai 200080, China; 2 Laboratory of Molecular Cell Biology, Institute of Biochemistry and Cell Biology, Cell Bank, Stem Cell Bank, Shanghai Institutes for Biological Sciences, Chinese Academy of Sciences, Shanghai 200031, China

**Keywords:** c-MYC, LIN28, KLF4, SOX2, OCT4, NANOG, Kidney Neoplasm

## Abstract

**Background:**

C-MYC, LIN28, OCT4, KLF4, NANOG and SOX2 are stem cell related factors. We detected whether these factors express in renal cell carcinoma (RCC) tissues to study their correlations with the clinical and pathological characteristics.

**Methods:**

The expressions of c-MYC, LIN28, SOX2, KLF4, OCT4 and NANOG in 30 RCC patients and 5 non-RCC patients were detected with quantitative real-time reverse transcription-PCR (qRT-PCR). The data were analyzed with Wilcoxon signed rank sum test and x^2 ^test.

**Results:**

In RCC group, c-MYC expression was significantly higher in RCC tissues compared with normal tissues (P < 0.05). The expression levels of OCT4, KLF4, NANOG and SOX2 were significantly lower in RCC tissues compared with normal tissues (P < 0.05). LIN28 expression level was not significant. No difference was observed when it comes to clinical and pathological characteristics such as gender, age, tumor size, cTNM classification and differentiation status (P > 0.05). Also the expression levels of all above factors were not significantly changed in non-RCC group (P > 0.05).

**Conclusions:**

The present analysis strongly suggests that altered expression of several stem cell related factors may play different roles in RCC. C-MYC may function as an oncogene and OCT4, KLF4, NANOG and SOX2 as tumor suppressors.

## Background

In 2007, Takahshi et al [1] successfully demonstrated the induction of pluripotent stem cells from adult human fibroblast by transfection of four transcription factors: OCT3/4, SOX2, c-MYC and KLF4. Yu et al [2] developed a similar method to restore the pluripotency of human somatic cells by transfecting OCT4, SOX2, NANOG and LIN28. Recently, we reported that transducting adult rat cells with lentivirus containing a cocktail of reprogramming factors of OCT4, SOX2, c-MYC and KLF4 could create rat pluripotent stem cell lines, rather than using lentivirus containing OCT4, SOX2, NANOG and LIN28 genes [3]. Besides the potential to induce the pluripotency, more studies are reported on cancer stem cells because of their functions to regulate the proliferation, differentiation and metastasis [4-10]. Bussolati et al [11] found tumor-initiating stem cells in human RCC; however, few studies combined all these stem cell related factors together in pathological specimens of RCC. RCC has increased over the last decades [12]. Our previous study on stem cells [3] triggered us to clarify the correlation between the clinical characteristics and the expressions of c-MYC, LIN28, KLF4, SOX2, OCT4 and NANOG in RCC, and thereby to evaluate their existence and roles in RCC.

## Methods

### Clinical samples of renal cell cancer

Totally, we collected the specimens of 35 patients after nephrectomy from December 2007 to October 2010 in Shanghai First People's Hospital. The specimens were collected from normal region (outside the range of tumor or nidus at least 5 cm macroscopically) and tumor/nidus for each patient. The case matched paired specimens were classified into the RCC group and non-RCC group according to the pathological examination. Finally 30 paired specimens identified as 28 clear cell carcinomas, 1 papillary carcinoma and 1 chromophobe renal carcinoma, were included in the RCC group, and 5 identified as 2 urothelial transitional cell cancer, 1 renal harmatoma, 1 renal tuberculosis and 1 benign tumor in the non-RCC group. In the RCC group there were 18 male patients and 12 female patients, with an average age of 60 (40 to 84). Of them 13 were classified as stage I, 13 as stage II, 4 as stage III in accordance with cTNM of AJCC (American Joint Committee on Cancer), whereas 6 as grade I, 15 as grade II and 9 as grade III according to their differentiation status. There were 4 female patients and 1 male patient in non-RCC group with the average age of 55.4 (44 to 68). Two patients diagnosed as urothelial neoplasms were classified as stage III and stage IV respectively according to cTNM classification and both of them were in grade I according to their differentiation status. All the specimens were kept at -80 °C immediately after surgery until RNA extraction. All patients were informed and consent. The tissue specimens were confirmed by a pathologist.

### Total RNA extraction, reverse transcription and qRT-PCR

Total RNA from tissues was extracted using TRizol reagent (Invitrogen, CA, cat.no: 15596-018). Briefly, total RNA was extracted from frozen tissues. The tissue was first ground into powder in mortar with continuous flow of liquid nitrogen to prevent the tissue from thawing. About 50-100 mg of the powder was homogenized in 1 mL TRizol reagent. The mixture was kept on ice for 5 min. After centrifuging at 16,200 ×g at 4°C for 15 min, the supernatant was transferred into another RNase-free tube along with 0.2 mL chloroform. The tube was shaken vigorously for 15 sec and kept on ice for 10 min. After centrifuging at 16,200 ×g at 4°C for 15 min, the supernatant was transferred into another tube with 0.5 mL isopropanol, blended and kept on ice for 5 min. After centrifuging at 16,200 ×g for 15 min g at 4°C for 10 min, the supernatant was discarded and the precipitate was washed with 75% ethanol at 4°C. After centrifuging at 5,600 ×g at 4°C for 6 min, the supernatant was discarded and the residue was air dried at room temperature. The RNA pellet was suspended with diethyl pyrocarbonate treated water at 55°C for 10 min.

RNA quantity and quality were determined by agarose gel electrophoresis and spectrophotometer at 260 nm.

Reverse transcription for mRNAs was performed using the PrimeScriptTM RT reagent kit (TaKaRa, Dalian, China, cat.no: DRR037A). The cDNA template was amplified by real-time PCR using the SYBR^® ^Premix EX TaqTM II kit (TaKaRa, Dalian, China, cat.no: DRR081A). Thermal cycling conditions were as follows: 95°C for 60 sec followed by 35 cycles of 94°C for 10 sec, 59°C for 30 sec and 72°C for 30 sec. After amplification, products were subjected to 60°C to 95°C, reading plate every 0.4°C and holding for 1 sec to create a melting curve. Glyceraldehyde-3-phosphate-dehydrogenase (GAPDH) mRNA was used as an internal control to normalize target mRNA level. PCR products were determined by electrophoresis on 1% agarose gels containing ethidium bromide.

Primer sequences are as follow: c-myc-forward, 5'-ACAGCTACGGAACTCTTGTGCGTA-3', reverse, 5'-GCCCAAAGTCCAATTTGAGGCAGT-3'; LIN28, forward 5'- GAAGAAGAAATCCACAGCCCTAC-3', reverse 5'- GATGGTGTGAACCCAAGCCTG-3'; SOX2, forward 5'- CCCATGCACCGCTACGACGTG-3', reverse 5'- GCTGGAGCTGGCCTCGGACTT-3'; KLF4, forward 5'- GAAATTCGCCCGCTCAGATGAACT-3', reverse 5'- TCTTCATGTGTAAGGCGAGGTGGT -3'; OCT4, forward 5'- TCCCATGCATTCAAACTGAGGTGC-3', reverse 5'- AACTTCACCTTCCCTCCAACCAGT-3'; NANOG, forward 5'- CCCAAAGGCAAACAACCCACTTCT-3', reverse 5'- AGCTGGGTGGAAGAGAACACAGTT-3'. GAPDH was used as an internal control with the following primers: forward 5'- TCGACAGTCAGCCGCATCTTCTTT -3', reverse 5'- ACCAAATCCGTTGACTCCGACCTT-3'.

The relative expression fold change of microRNAs and target mRNAs were calculated by the 2^-ΔΔCt^ method [13]. All reactions were performed in triplicate.

### Statistical Analysis

All tests were analyzed by SAS 8.0 with Wilcoxon signed rank sum test and x^2 ^test. P-value < 0.05 was considered to be statistically significant.

## Results

### Deregulation of c-MYC, LIN28, OCT4, KLF4, NANOG and SOX2

qRT-PCR analysis demonstrated that in the RCC group the expressions of stem cell related factors except LIN28 in cancer tissues were significantly changed comparing with normal tissues (P < 0.05)( Table 1). C-MYC was significantly up-regulated and KLF4, SOX2, OCT4, and NANOG were significantly down-regulated (Table 1). However, the expressions showed no significance in the non-RCC group (data not shown).

**Table 1:** Significances of deregulated stem cell related factors


**names**	**No of down regulated**	**No of up regulated**	**P value**

**C-MYC**	8	22	0.0010
**LIN28**	15	15	0.6083
**KLF4**	25	5	0.0050
**SOX2**	28	2	< 0.0001
**OCT4**	26	4	0.0070
**NANOG**	29	1	< 0.0001

The data were from RCC group. In each paired case matched specimens (cancer and normal tissue from the same patient), the expression level of cancer comparing with normal tissue more than 1 is considered up regulated, or it is down regulated. All experiments are performed three times. P value < 0.05 was considered statistically significant

### The association between expression level with clinical and pathological characteristics

We analyzed the data to see if any correlation exists between the stem cell related factors and clinical characteristics. No expression significance of each stem-cell related factor in gender, age and tumor size was observed between RCC and non-RCC group (Table 2, 3).

**Table 2:** The expression levels of stem cell related factors in terms of clinical and pathological characteristics


**Items**	**No of cases**	**C-MYC**		**LIN28**		**KLF4**		**SOX2**		**OCT4**		**NANOG**	
		
		↑	↓	↑	↓	↑	↓	↑	↓	↑	↓	↑	↓

**Gender**													
**Male**	18	11	7	11	7	3	15	2	16	3	15	1	17
**Female**	12	11	1	4	8	2	10	0	12	1	11	0	12
**Age (years)**													
**≤65**	22	17	5	12	10	4	18	2	20	4	18	1	21
**>65**	8	5	3	3	5	1	7	0	8	0	8	0	8
**Tumor size (cm)**													
**≤4**	12	9	3	7	5	3	9	1	11	3	9	1	11
**>4**	18	13	5	8	10	2	16	1	17	1	17	0	18
**cTNM**													
**I**	13	10	3	8	5	2	11	1	12	2	11	1	12
**II**	13	9	4	6	7	2	11	1	12	1	12	0	13
**III**	4	3	1	1	3	1	3	0	4	1	3	0	4
**Differentiation**													
**Well**	6	5	1	3	3	1	5	0	6	0	6	0	6
**Moderate**	15	12	3	7	8	3	12	2	13	2	13	1	14
**Poor**	9	5	4	5	4	1	8	0	9	2	7	0	9

The data were from RCC group. ↑represent in each paired case matched specimens (cancer and normal tissue from the same patient), the expression level of cancer comparing with normal tissue is more than 1, or it is down regulated and it is represented by ↓. All experiments are performed three times.

**Table 3:** Significances of deregulated stem cell related factors in terms of clinical and pathological characteristics


**Items**	**P value**				
	
	**Gender**	**Age**	**Tumor size**	**cTNM**	**Differentiation**

**C-MYC**	0.0683	0.4263	0.8684	0.8141	0.2020
**LIN28**	0.1427	0.4169	0.4637	0.1936	0.7976
**KLF4**	1.0000	0.7166	0.3255	0.7272	0.7308
**SOX2**	0.2399	0.3855	0.7689	0.6767	0.8371
**OCT4**	0.5178	0.2028	0.1313	0.8784	0.2274
**NANOG**	0.4142	0.5465	0.2207	0.3106	0.8864

The data were from RCC group. Expression levels were combined with clinical and pathological characteristics with Wilcoxon signed rank sum test and x^2 ^test. P value < 0.05 was considered statistically significant.

As for the pathological characteristics such as cTNM classification and the differentiation status of cancers, the expression levels of c-MYC, LIN28, KLF4, SOX2, OCT4 and NANOG were not significant either( Table 2, 3)

## Discussion

Although the number of the RCC cases was limited in our study, the results are still with interesting implications. First, in the RCC group the over expression of c-MYC in cancers was significantly higher than that of normal tissues (Table 1). No difference of c-MYC was observed in the non-RCC group. This is in accordance with Tang's report [4], and c-MYC is an oncogene associated with RCC growth and proliferation by up regulating target genes like BCL2, CCND1, PCNA, PGK1 and VEGFA. C-MYC could also induce a somatic cell to regain pluripotency together with OCT4, SOX2 and KLF4 [1]. In addition, c-MYC can activate the transcription of hTERT that can regulate the activation of telomerase [14]. These all indicate that c-MYC plays an important role in RCC.

Interestingly an important regulatory network involving c-MYC, let-7 and LIN28/L28b was found recently [6,15,15-23]. There is a double-negative-feedback loop between LIN28/IN28b and let-7 where let-7 is able to repress LIN28/LIN28b by binding to the 3'UTR of LIN28/LIN28b transcripts and LIN28/LIN28b can also repress let-7 [15-17]. Another feedback loop is between LIN28 and c-MYC: LIN28/LIN28b up regulates c-MYC by repressing let-7 [6] and c-MYC transcriptionally activates both LIN28 and LIN28b [18]. Also, c-MYC is one of the targets of let-7 [19-21]; in return let-7 can be repressed by MYC contributing to tumorigeness [22,23]. Besides let-7, derepression of c-MYC might also contribute to the repression and activation of diverse miRNAs related to tumorigeness [22]. The whole regulatory circuits may contribute to the widespread deregulation of miRNAs in many human malignancies [24]. Although the expression of LIN28 was not significantly different between normal specimens and tumors and we see no expression difference of LIN-28 in non-RCC group either (Table 1), we think that we need to do more to explore the function of LIN28/let-7/c-MYC regulatory circle in RCC.

KLF4 is a member of Kruppel like factors, which has a dual function related to the tumorigeness [25]. KLF4 plays as a tumor suppressor in gastric cancer, colon cancer, esophagus cancer, bladder cancer, lung cancer and pancreatic ductal carcinoma [7,26-28]. Our study shows that the expression of KLF4 in cancers was significantly lower than that in normal tissues (Table 1). This suggests that KLF4 may function as a tumor suppressor in RCC.

Lu [10] reported that SOX2 was over-expressed in human squamous cell lung tumors and some adenocarcinomas. Sanada et al [29] saw an increasing expression of SOX2 with the progression of pancreatic carcinoma. In our study, SOX2 was lower in cancers compared with normal tissues. Its expression level was significantly down regulated in RCC (Table 1). Given that some biomarker like KLF4 mentioned above may function totally reversely in different cancers [25], we think it is possible that SOX2 may be a tumor suppressor in RCC.

OCT4 is essential to reprogram somatic ells to regain pluripotency [30]. Looijenga [31] found that OCT4 is positive in RCC, but its expression is quite low comparing with germ cell tumor. So it is in accordance with the theory that the amount of cancer stem cell is quite small in tumor and even less in solid tumor [32,33]. As for NANOG, Yu [2] achieved a significant breakthrough in reprogramming somatic cells back to pluripotent stem cells. As reported by Ezeh [8] and Hart [9], NANOG expressed in embryonal carcinomas, seminomas and breast cancer, and could be a valuable marker of tumorigeness. According to the report of Bussolati et al [11], the RCC stem cells they identified were OCT4 and NANOG sharply positive, but we saw some interesting results about the above-mentioned two factors, they both were significantly down regulated in RCC (Table 1). Also we did not see any significant correlation between their expression and clinical and pathological characteristics (Table 2, 3). We thought there were several reasons contributing to this. First, OCT4 and NANOG are not specific biomarkers for RCC or they do not play dominant roles in the tumorigeness of RCC, but according to Bussolati's [11] report, OCT4 and NANOG are expressed in tumor-initiating CD105+ stem cells in human renal carcinomas. Cells from CD105+ clones cultured in epithelial or endothelial differentiating medium for 2 weeks acquired the expression of differentiative markers and lost stem cell markers. So this may not be the main reason for our result. Second, most cells in cancer are subgroups of cancer stem cells which only express a narrow range of stem cell related factors. So only a tiny part of these cells can present the whole characters of stem cell. Last but not the least, the cancer stem cell is really rare in the tumor as mentioned previously [32,33]. Also, OCT4 and NANOG might function as tumor suppressors in RCC indeed. We think Bussolati's [11] positive findings of OCT4 and NANOG in RCC stem cells is because that they isolated CD105+ cells from other ones just like a kind of purification. According to Moreira's [34] report, in lung cancers, stem cell markers are expressed with different patterns seen for different histological types and degrees of differentiation, considering that our RCC specimens were restricted to clear cell RCC (28 in 30), we still cannot eliminate OCT4 and NANOG as non-potential biomarkers for RCC and more comprehensive studies are needed to elucidate their importance.

Although all stem cell related factors except LIN28 were significantly deregulated (Table 1), there were no significant results in terms of clinical and pathological characteristics (Table 2, 3). There are some reasons contributing to this. Fist, we think that if cancer stem cells do exist in RCC, they could just initiate the carcinogenesis progress, and were not dependable after initiation. Second, our patient number is limited and we don't have follow-up information. Like Zhang's [35] report about OCT4 in lung adenocarcinoma, there were no significant findings in terms of clinical and pathological characteristics, but when they combined OCT4 expression level with the follow-up information of 5 years, OCT4 was found to be the independent prognostic factor. So this may be one of drawbacks in our study. But our study first combined all the six stem cell related factors with tissue specimens. This will provide us with useful information to explore more about the function of the six stem cell related factors in RCC.

## Conclusions

In conclusion, the results of our analysis of stem cell related factors between RCC and non-RCC groups, suggest that deregulation of these factors may be one of the oncogenic mechanisms underlying RCC pathogenesis. Especially, the expression of c-MYC, KLF4, OCT4, NANOG and SOX2 implies that these 5 factors are involved in carcinogenesis and progression of RCC, and probably there are RCC cancer stem cells existing in renal carcinoma. More data are needed to confirm our hypothesis.

## Abbreviations

RCC: renal cell carcinoma; qRT-PCR: quantitative real-time reverse transcription-PCR; GAPDH: glyceraldehyde-3-phosphate-dehydrogenase.

## Competing interests

The authors declare that they have no competing interests.

## Authors' contributions

YL and CZ carried out all the experimental studies. YL drafted the manuscript and performed the statistical analysis. BY and LZ collected all specimens. JF conceived of the study and participated in its design and coordination. JF, LX and SX helped to draft the manuscript. All authors read and approved the final manuscript.
